# Egg Parasitoids of Proconiini (Hemiptera: Cicadellidae) in Northwestern Mexico, with Description of a New Species of *Gonatocerus* (Hymenoptera: Mymaridae)

**DOI:** 10.1673/031.009.0501

**Published:** 2009-03-17

**Authors:** Serguei V. Triapitsyn, Julio S. Bernal

**Affiliations:** ^1^ Entomology Research Museum, Department of Entomology, University of California, Riverside, California, 92521, USA; ^2^ Biological Control Laboratory, Department of Entomology, Texas A&M University, College Station, Texas, 77843-2475, USA

**Keywords:** Biological control, Trichogrammatidae, *Homalodisca vitripennis*, *Burksiella*, *lttys*, *Pseudoligosita*, *Oncometopia*

## Abstract

Nine species of Mymaridae and Trichogrammatidae parasitic on eggs of Proconiini sharpshooters (Cicadellidae: Cicadellinae) were collected in northwestern Mexico in relation to neoclassical biological control efforts against glassy-winged sharpshooter, *Homalodisca vitripennis* (Germar), in California. *Gonatocerus chula* Triapitsyn and Bernal sp. n., which belongs to the *ater* species group of *Gonatocerus* Nees (Mymaridae), is described. Specimens of *G. chula* sp. n. were reared from eggs of the smoke-tree sharpshooter, *Homalodisca liturata* Ball, on jojoba [*Simmondsia chinensis* (Link) C. K. Schneider] leaves collected in central Sonora state, Mexico. Also given are new data on other egg parasitoids of *Homalodisca* spp. and *Oncometopia* spp. in Sinaloa and Sonora states, Mexico, including *Gonatocerus atriclavus* Girault, *G. morrilli* (Howard), and *G. novifasciatus* Girault, and the Trichogrammatidae *Burksiella* sp(p)., *Ittys* sp., *Pseudoligosita* sp., *Ufens ceratus* Owen, and *U. principalis* Owen. For the first time, a species of *Ittys* is recorded from eggs of Proconiini, and *U. principalis* from Mexico. Colonies of *G. atriclavus*, *G. novifasciatus* and *Pseudoligosita* sp. were successfully established in a quarantine laboratory at University of California, Riverside, on eggs of the glassy-winged sharpshooter. These three parasitoid species had never been reared under laboratory conditions. In addition, seven species of Proconiini were collected in central and northwestern Mexico: *Cyrtodisca major* (Signoret), *Homalodisca insolita* (Walker), *H. liturata* Ball, *Oncometopia* sp. cf. *clarior* (Walker), *O*. sp. cf. *trilobata* Melichar, *O*. (*Similitopia*) sp., and *Phera centrolineata* (Signoret). *Oncometopia* sp. cf. *clarior*, *O*. sp. cf. *trilobata*, and *O*. (*Similitopia*) sp. appeared to be undescribed species.

## Introduction

Egg parasitoids within the families Mymaridae and Trichogrammatidae (Hymenoptera) are commonly hosted by Proconiini sharpshooters (Hemiptera: Cicadellidae: Cicadellinae: Proconiini), such as the glassy-winged sharpshooter, *Homalodisca vitripennis* (Germar). *H. vitripennis*, among the potentially most destructive exotic pests to ever enter California, is native to the southeastern USA and northeastern Mexico, and became established in that state ca. 1990 (Sorensen and Gill 1996). The economic importance of *H. vitripennis* stems mostly from its efficiency as a vector of the plant pathogenic bacterium *Xylella fastidiosa* Wells, Raju, Hung, Weisburg, Mandelco-Paul, and Brenner, which is the causal agent of Pierce's disease in grapes, among other important diseases.

Substantial research emphasis has been placed on importation biological control of *H. vitripennis*. The exotic origin of *H. vitripennis*, and a long history of successful biological control efforts against numerous exotic pests in California indicated that importation biological control of *H. vitripennis* could be successful in permanently reducing the abundance of this pest in that state. Importation biological control of *H. vitripe*nnis in California has focused on importing parasitoid species from the southeastern USA, and to lesser extents from northeastern and southeastern Mexico, midwestern USA, and Argentina. To date, at least five species of Mymaridae egg parasitoids have been imported and released in California; all were introduced from the southeastern USA or northeastern Mexico, except *Anagrus epos* Girault, which was imported from Minnesota (Triapitsyn and Phillips 2000; Triapitsyn and Hoddle 2001, 2002; Triapitsyn et al. 2002; Hoddle and Triapitsyn 2004; Pilkington et al. 2005; Triapitsyn 2006a, b; Triapitsyn et al. 2006). In addition to the species released to date, several others have been imported from Argentina (where *H. vitripennis* and other *Homalodisca* spp. are not known to occur), and northeastern Mexico, though they have not been released (Logarzo et al. 2004; Hoddle and Triapitsyn 2005). Thus, two approaches to importation biological control of *H. vitripennis* in California are being followed. The classical approach of reuniting an exotic pest, such as *H. vitripennis*, with its coevolved natural enemies from the pest's area of origin is being explored via importation of parasitoids from the southeastern USA and northeastern Mexico. The neoclassical approach of importing non-coevolved natural enemies (i.e. parasitic on closely related host species or genera) against exotic pests is being explored via importation of parasitoids from Minnesota, southeastern Mexico (where the occurrence of *H. vitripennis* is uncertain), and Argentina.

The long-term goal of the activities described herein was to contribute to neoclassical biological control efforts against *H. vitripennis*, through importation of natural enemies from central and northwestern Mexico. At least six species of Proconiini were known from those areas, though it was doubtful that *H. vitripennis* occurred there (MacGregor and Gutiérrez 1983; Pacheco Mendivil 1985; Takiya 2006; R. A. Rakitov, personal communication). Neoclassical biological control efforts have been successful in a number of outstanding successful cases of importation biological control (Wiedenmann and Smith 1997; Legner and Bellows 1999). A neoclassical approach against *H. vitripennis* was especially promising because efforts to date have not uncovered natural enemies in the pest's native range that effectively suppress the first (spring) generation of *H. vitripennis* in California, and because closely related host species, that may share physiological and ecological characteristics with *H. vitripennis*, occur in central and northwestern Mexico. This report presents the results of activities aimed at surveying and collecting egg parasitoids of Proconiini in the Mexican states of Colima, Jalisco, Nayarit, Sinaloa, and Sonora between July, 2006, and July, 2007.

## Material and Methods

### Collecting and shipping of material

An exploratory collecting trip was made between 10 July and 7 August, 2007. The primary goal of this trip was to identify prime sites and host plants for subsequent collection of parasitized Proconiini egg masses for shipment to California. Generous hands-on assistance during collections, as well as assistance in trip coordination and planning were provided by colleagues (and their assistants) based along the trip's route: Dr. Gustavo Moya Raygoza (Universidad de Guadalajara, Zapopan, Jalisco), Dr. Edgardo Cortez Mondaca (Instituto Nacional de Investigaciones Forestales, Agrícolas, y Pecuarias, Juan José Rios, Sinaloa), and Mr. Agustín Fú Castillo (Instituto Nacional de Investigaciones Forestales, Agrícolas, y Pecuarias, Hermosillo, Sonora). The exploratory trip was initiated in Guadalajara, Jalisco, and continued southwest along Highways MEX 15 and MEX 80 to Melaque, Jalisco. From Melaque, the trip continued east to Manzanillo, Colima, along MEX 200, then north along MEX 200D, MEX 110, MEX 54D, and MEX 15, to the starting point in Guadalajara. From Guadalajara, the trip continued northwest along MEX 15 through the states of Nayarit and Sinaloa, and to Hermosillo, in central Sonora state. In addition, this trip included numerous minor roads (paved and unpaved, mapped and unmapped) intersecting the main highways listed above. A total of ∼,300 km were traveled by road between 10 July and 7 August, 2007. Collecting methods during this trip included sweeping vegetation, visual observation and hand collection from host plants, and light trapping with 250 watt mercury vapor lights against white cotton bed sheets. In addition, yellow sticky card traps deployed in a local whitefly monitoring network in and around Los Mochis, Sinaloa, were examined for adult Proconiini (n = 254 traps; 26–52 traps in each of July, September, and December, 2006, and March, and May, 2007). Additional sticky card traps (n = 42 traps, 18 and 24 traps in July, 2006, and May, 2007, respectively) were examined in Zapopan, Jalisco (May, 2006, May 2007), where they had been deployed in an experimental maize field. These sticky card traps greatly facilitated discovery of Proconiini in the field. Sites and host plants where Proconiini adults were discovered were searched immediately for egg masses, and recorded for subsequent searching. Subsequent searches in selected sites (4 or 5 days each, including travel to and from the sites) were made in September, November, and December, 2006, and March, May, June (12 days), and July, 2007. Sample specimens of all Proconiini adults found were sent to Dr. Roman A. Rakitov (Illinois Natural History Survey, Champaign, Illinois, USA) for identification and vouchering.

Upon discovery, Proconiini egg masses were collected along with a small portion of the plant substrate on which they were found (typically a leaf), inspected visually with a magnifying loupe (10×) in the field to eliminate egg masses from which parasitoids had already emerged, and placed in a cooler until they could be stored in a refrigerator (∼10°C), or packaged for shipping to the quarantine facility at the Department of Entomology, University of California, Riverside, California, USA (hereafter UCR). Packaging consisted of placing the corresponding plant material with egg masses in plastic containers with a cotton fabric window for aeration, and the plastic containers inside Ziploc® plastic bags (www.ziploc.com). The plastic bags were then shipped to the quarantine facility at UCR in a styrofoam cooler with Cold-Ice® gel refrigerant (www.coldice.com). All egg mass shipments were sent under the appropriate federal permit to the UCR quarantine facility via the designated United Stated Department of Agriculture inspection stations.

### Quarantine work

Upon arrival at the UCR quarantine facility, the shipments were processed according to current federal and state regulations, and assigned UCR quarantine Shipping and Receiving (S&R) numbers. Then, the leaves with the parasitized egg masses of the host sharpshooter were segregated inside sealed plastic bags, and the bags were placed inside sealed emergence cages. The samples were examined daily for emergence. Any emerged parasitoids (including adult parasitoids emerging en route to UCR) were collected into sealed glass vials, and identified at least to genus under a dissecting microscope within the quarantine laboratory. If sufficient parasitoids were available, a few voucher specimens were taken by placing them in 95% ethanol. Voucher specimens were point- or slide-mounted outside of the quarantine facility, if necessary, to confirm the identifications. Some common egg parasitoids of Proconiini, such as *Gonatocerus morrilli* (Howard) (Mymaridae), which had already been introduced into California (de León and Morgan 2007), or *Ufens ceratus* Owen and *U. principalis* Owen (Trichogrammatidae), which naturally occur in California (Al-Wahaibi et al. 2005), were not propagated and were placed directly in 95% ethanol as vouchers. *Burksiella* spp. (Trichogrammatidae) were recovered, but were not propagated because of the known difficulties associated with their rearing under laboratory conditions (Triapitsyn 2003), and the resulting low interest in them as potential biological control agents. All other parasitoids (when both genders were available, these were placed in the same vial for at least several hours) were exposed to eggs of *H. vitripennis* on *Euonymus japonica* Thunberg (Celastraceae) leaves in small cages maintained at 22–23°C. After several days, when initial signs of parasitization became apparent (i.e., the host eggs became dark), the colony founders were removed from the cages and preserved in ethanol as vouchers. Two to five days following the emergence of F1 parasitoid adults, they were offered freshly laid *H. vitripennis* egg masses for further reproduction.

### Parasitoid identification and taxonomic work

All determinations were done by SVT using point- and slide-mounted specimens, or specimens in ethanol. In most cases, the trichogrammatids were identified to genera only because of the lack of suitable keys; *Ufens* spp. were identified following Al-Wahaibi et al. (2005). *Gonatocerus* is a well-known genus, and its generic and species group diagnoses are available elsewhere (Huber 1988). Terminology for the morphological features is that of Gibson (1997). All voucher specimens were deposited in the Entomology Research Museum, University of California, Riverside (hereafter UCRC). An abbreviation used in the taxonomic description below is “F” for antennal funicular segment (of females) or antennal flagellar segment (of males).

## Results and Discussion

### Proconiini collections

Adult specimens of Proconiini species were collected and identified from the states of Colima, Jalisco, Sinaloa, and Sonora, between July, 2006, and July, 2007. *Cyrtodisca major* (Signoret) and *Homalodisca insolita* (Walker) were collected in Colima; *C. major*, *H. insolita*, *Oncometopia* sp. cf. *trilobata* Melichar, and *Phera centrolineata* (Signoret) were collected in Jalisco; *Oncometopia* (*Similitopia*) sp. and *O*. sp. cf. *clarior* (Walker) were collected in Sinaloa; and *Homalodisca liturata* Ball was collected in Sonora. Adult Proconiini were not collected in Nayarit. *Oncometopia* sp. cf. *clarior*, *O*. sp. cf. *trilobata*, and *O*. (*Similitopia*) sp. appeared to be undescribed species (R. A. Rakitov, pers. comm.). Precise distributional and host plant records for these Proconiini will be presented separately. Vouchers of these specimens were deposited in the Insect Collection of the Illinois Natural History Survey, Champaign, Illinois.

**Figures 1–4.**
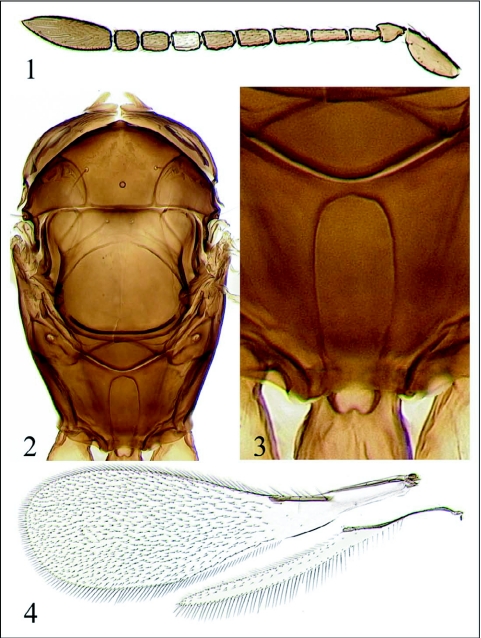
*Gonatocerus chula* sp. n. (female, holotype). 1. Antenna. 2. Mesosoma. 3. Propodeum. 4. Wings.

Previously, six species of Proconiini had been reported from the states of Colima, Jalisco, Sinaloa, and Sonora, viz. *Cuerna angusta* Nielsen, *C. major*, *Homalodisca ichthyocephala* (Signoret), *H. insolita*, *H. liturata*, and *Phera aterrima* Fowler (MacGregor and Gutiérrez 1983; Pacheco Mendivil 1985; Takiya 2006; R. A. Rakitov, pers. comm.). Thus, collections between 2006 and 2007 added four new records to the known Proconiini fauna of those states: *Oncometopia* sp. cf. *clarior*, *O*. sp. cf. *trilobata*, *O*. (*Similitopia*) sp., and *P. centrolineata*.

Proconiini egg masses were shipped to UCR from Sinaloa and Sonora, and these were of *Oncometopia* (*Similitopia*) sp. or *H. liturata*, respectively. Data on host plants, and collection localities and dates for these egg masses, and corresponding species, are provided below. Egg masses were not found in the states of Jalisco, Colima, and Nayarit.

### Taxonomy

**Figures 5, 6.**
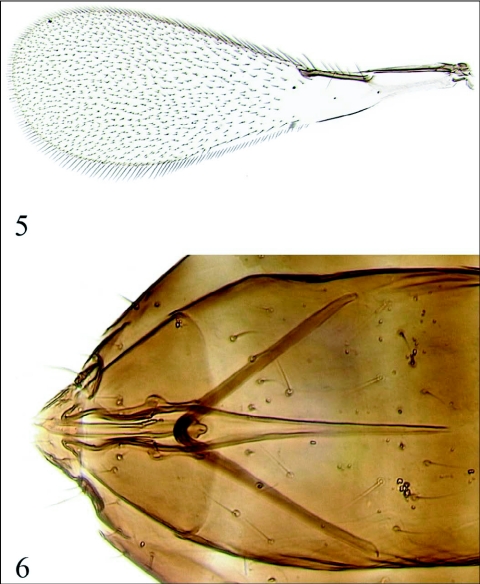
*Gonatocerus chula* sp. n. (male, paratype). 5. Forewing. 6. Genitalia.

***Gonatocerus chula* Triapitsyn and Bernal, sp. n. (Figures 1–6)**
**Type material**
  Holotype female on slide [UCRC]: MEXICO, Sonora, “Campo Nuevo” (nr. Miguel Alemán, 28°50′15″N 111°28′25″W), 2.iv.2007, J. Bernal, emerged 12.iv.2007 in UCR (Riverside, California, USA) Quarantine (S&R 07–19, coll. by V. Berezovskiy) from eggs of *Homalodisca liturata* Ball on jojoba leaves. Paratype: same data as the holotype, except emerged 13.iv.2007 [1 male on slide, UCRC].
Description FEMALE (holotype)
  Head and mesosoma dark brown to black, metasoma brown; borders of face, parts of vertex, radicle, and F6 yellow to light brown; procoxa mostly yellow, remainder of leg segments light brown except metatibia, and all tarsi brownish.
  **Antenna** (Figure 1) with radicle 2.3× as long as wide, scape 2.6–2.7× as long as wide, very finely longitudinally striate; pedicel slightly longer than F1; F1–F4 slightly narrower than F5–F8, F2 longest, F8 shortest of funicular segments; F1 without sensilla, F2 with 1 longitudinal sensillum on one antenna, but without such sensillum on the other antenna, F3 with 1 longitudinal sensillum, F4–F8 each with 2 longitudinal sensilla; all funicular segments densely setose; clava with 8 longitudinal sensilla, 3.1× as long as wide, shorter than combined length of F1–F3, its ventral surface covered with numerous minute, short setae, and placoid sensilla, its dorsal surface densely covered with longer setae.
  **Mesosoma** (Figure 2). Pronotum lightly reticulate; mesoscutum, scutellum, axillae, and dorsellum almost smooth. Each lobe of pronotum with 2 strong dorsal and 2 weak lateral setae. Mesoscutum wider than long, shorter than scutellum; midlobe of mesoscutum with a pair of strong adnotaular setae. Dorsellum rhomboidal. Propodeum (Figure 3) with well-developed lateral and submedial carinae, smooth between submedial carinae and almost smooth between submedial and lateral carinae (except distally), and slightly wrinkled lateral to lateral carinae. Pro tibia without conical sensilla. Wings (Figure 4) hyaline. Forewing 3.2× as long as wide; marginal setae short, the longest marginal seta about 1/6 maximum wing width. Forewing blade bare behind venation, except for 1 seta behind apex of marginal vein, remainder of blade densely setose. Submarginal vein with 1 macrochaeta and 2 microchaetae, marginal vein with 4 strong setae between proximal and distal macrochaetae; hypochaeta closer to distal macrochaeta than to proximal macrochaeta. Hind wing 15× as long as wide; the longest marginal seta 1.5–1.6× maximum wing width; blade mostly bare except for complete rows of setae along margins and a submedian row of discal setae beyond tip of venation.
  **Metasoma.** Petiole slightly longer than wide, trapezoidal. Ovipositor about 2/3 length of gaster, barely exserted beyond its apex. Ovipositor:mesotibia ratio 1.1:1; ovipositonmetatibia ratio 1.0:1. Outer plates of ovipositor each with 1 distal seta.
  Measurements (in µm, as length or length:width [for the wings only]). Body (taken from critical point dried specimen before slide-mounting): 1453; head (taken from critical point dried specimen before slide-mounting): 215; mesosoma 621; petiole 98; gaster 738; ovipositor 506. Antenna: radicle 36; rest of scape 158; pedicel 70; F1 67; F2 88; F3 85; F4 83; F5 80; F6 70; F7 68; F8 58; clava 215. Forewing 1507:467; longest marginal seta 79. Hind wing 1150:76; longest marginal seta 118.
**MALE (paratype)**
  Body length (taken from slide-mounted specimen) 1660 µm. Similar to female in coloration except vertex all dark brown to black, antenna brown. Antenna with scape about 2.8× as long as wide; pedicel small, F1 a little shorter than following flagellar segments, F2–F11 more or less subequal in length, all flagellomeres with 10 to 12 longitudinal sensilla. Forewing (Figure 5) 2.9× as long as wide; hind wing about 13× as long as wide. Genitalia (Figure 6) typical of the *morrilli* subgroup of the *ater* species group of *Gonatocerus*; apex of apodeme of genital sternite acute.
**Etymology**
  The specific name is a noun in apposition; it is a Mexican colloquial noun used as an affected form of address.
**Diagnosis**
  The dark body color and the contrastingly light F6 of the female antenna distinguishes *G. chula* sp. n. from the other described Nearctic species from the *morrilli* subgroup of the *ater* group of *Gonatocerus*, except *G. morgani* Triapitsyn, described from southern California, USA (Triapitsyn 2006a). The new species differs from *G. morgani* primarily by the darker F5 of the female antenna, and especially by the submedial carinae of the propodeum [very thin and meeting basally in *G. chula* sp. n. (Figure 3), but very thick and not meeting basally in *G. morgani*, as illustrated by Triapitsyn (2006a)], and also the sculpture of the propodeum (almost smooth between the submedial and lateral carinae in *G. chula* sp. n., but notably wrinkled in *G. morgani*). Also, the female antenna of *G. chula* sp. n. at most has 1 longitudinal sensillum on F3, whereas that of *G. morgani* has 2 such sensilla on F3. *Gonatocerus chula* sp. n. does not match the descriptions and types of any of the numerous species of *Gonatocerus* from Argentina and elsewhere in Central and South America described by A. A. Ogloblin and others [S.V.T. examined all of them (except for one lost type of an unrelated species from Ecuador, which belongs to the *membraciphagus* species group) for the forthcoming revision of the described Neotropical species of *Gonatocerus* (Triapitsyn 2006b)].

### Host

*Homalodisca liturata* Ball

#### Notes on other egg parasitoids of Proconiini from northwestern Mexico Mymaridae

##### Gonatocerus atriclavus Girault

Material examined: MEXICO, Sinaloa, El Guayabo (nr. Ahome, 25°56′07″N 109°08′47″W, 11 m), 17.vii.2007,J. Bernal, emerged 23–26.vii.2007 in UCR quarantine from eggs of *Oncometopia* (*Similitopia*) sp. on leaves of an unknown plant and *Conyza canadensis*, S&R # 07–31 [8 females, 3 males, UCRC]. Additional 4 females and 4 males came in alive on 23.vii.2007 in the same shipment to UCR quarantine (under S&R # 07-31, these served as colony originators), of which 1 male was preserved as a voucher [UCRC]. Also vouchered were 5 females and 6 males [UCRC] of the first generation progeny of the aforementioned original wasps (emerged 11.viii.2007).

###### Comments

This species was recently redescribed, diagnosed, and illustrated by Triapitsyn et al. (2002). Triapitsyn (2006a) provided updated information on its distribution and host associations. This is the first time that *G. atriclavus* was reared under laboratory conditions (on eggs of *H. vitripennis* on *E. japonica* leaves). At the time when this communication was written (late September 2007), the colony in UCR quarantine successfully reached the F3 generation (the developmental period from exposure to the host eggs to emergence of the next generation wasps was 18 to 19 days at 22–23°C). Earlier attempts to rear *G. atriclavus* under quarantine laboratory conditions on *H. vitripennis* eggs were not successful (Triapitsyn et al. 2002). *Gonatocerus atriclavus* is a solitary parasitoid, producing one adult per each host egg. All specimens recovered from the El Guayabo, Sinaloa, site emerged from egg masses of *Oncometopia* (*Similitopia*) sp. collected from weeds (*Helianthus annuus* L., *Conyza canadensis* (L.) Cronquist, *Amaranthus* sp., one unknown species) in the vicinity of *Lantana* sp. shrubs. The *Oncometopia* (*Similitopia*) sp. host appears to be an undescribed species. Adults of *Oncometopia* (*Similitopia*) sp. were initially found and particularly abundant on *Lantana* sp., in July, 2006, at El Realito, Sinaloa (26° 22′ 95″ N 108° 41′ 29″ W, 76 m), and subsequently at El Guayabo, Sinaloa, though egg masses were not found on this host plant after searching in one or both localities in July, September, and December, 2006, and March, May, June, and July, 2007; egg masses were only recovered from surrounding weeds (noted above) at the El Guayabo site. Thus, *Lantana* sp. appears to be favored by *Oncometopia* (*Similitopia*) sp. for feeding, but not for oviposition. All *Lantana* sp. and weed hosts of *Oncometopia* (*Similitopia*) sp. were in the vicinity of a permanent source of water, such as an irrigation ditch or a river.

##### *Gonatocerus morrilli* (Howard)

Material examined: MEXICO, Sonora, “Campo La Esperanza” (nr. Miguel Alemán): 10.vi.2007, J. Bernal (emerged 26.vi.2007 in UCR quarantine from eggs of *Homalodisca liturata* on leaves of *H. annuus*, S&R # 07–26) [2 females, UCRC]; 2.vii.2007, A. Fú-Castillo (ex. eggs of *Homalodisca liturata* on leaves of *C. canadenis*, came in dead to UCR quarantine, S&R # 07–29) [2 females, 5 males, UCRC].

###### Comments

This mymarid species was recently diagnosed and illustrated by Triapitsyn (2006a), who also provided information on its taxonomic history, distribution, and host associations. It is a common, solitary egg parasitoid of various Proconiini in southern USA, and Mexico.

##### *Gonatocerus novifasciatus* Girault

Material examined: MEXICO, Sonora, “Campo Nuevo” (nr. Miguel Alemán), 3.iv.2007, J. Bernal (emerged 13.iv.2007 in UCR quarantine from eggs of *Homalodisca liturata* on leaves of jojoba, *Simmondsia chinensis*, S&R # 07–19) [3 females, 4 males, UCRC].

###### Comments

This species was recently diagnosed and illustrated by Triapitsyn (2006a), who also provided information on its taxonomic history, distribution, and host associations. It is a common egg parasitoid of Proconiini in the USA (including California), and several other countries, including Mexico. *G. novifasciatus* was reared for the first time under laboratory conditions (on eggs of *H. vitripennis* on *E. japonica* leaves).The colony in UCR quarantine was discontinued 4.vi.2007 after only males were obtained in the F2 generation (the F1 generation included both sexes); 25 of these males were preserved as vouchers [UCRC]. *Gonatocerus novifasciatus* is a solitary parasitoid.

##### Trichogrammatidae *Burkslella* sp(p).

Material examined: MEXICO: Sinaloa, El Guayabo (nr. Ahome), 17.vii.2007, J. Bernal (emerged 23–26.vii.2007 in UCR quarantine from eggs of *Oncometopia* (*Similitopia*) sp. on leaves of an unknown plant, S&R # 07–31) [10 females, 3 males, UCRC] (see above, *G. atriclavus*, for additional information on host plants). Sonora: “Campo La Esperanza” (nr. Miguel Alemán), 2.vii.2007, A. Fú-Castillo, received 10.vii.2007 (emerged en route to UCR quarantine ex. eggs of *Homalodisca liturata* on leaves of *C. canadensis*, S&R # 07–29) [numerous females and males, UCRC].

###### Comments

This (or these) trichogrammatid species are members of the poorly known genus *Burksiella* De Santis. Some species in this genus [the known egg parasitoids of Proconiini (Triapitsyn 2003)] were previously placed in the genus *Zpgella* Girault (Pinto 2006).

##### *lttys* sp.

Material examined: MEXICO, Sonora, “Campo La Esperanza” (nr. Miguel Alemán), 2.vii.2007, A. Fú-Castillo (emerged 24.vii.2007 in UCR quarantine ex. eggs of *Homalodisca liturata* on leaves of *C. canadensis*, S&R # 07–29) [2 females, 1 male, UCRC].

###### Comments

This is a distinctive, undescribed species, which is to some extent similar to *Ittys ceresarum* (Ashmead) (J. N. George, personal communication). The present is the first record of an *Ittys* Girault species from eggs of Proconiini. Other North American representatives of *Ittys* are known from eggs of Cicadidae, Membracidae, and Miridae (George 2007).

##### *Pseudoligosita* sp.

Material examined: MEXICO, Sonora, “Campo La Esperanza” (nr. Miguel Alemán), 2.vii.2007, A. Fú-Castillo (arrived to UCR quarantine 10.vii.2007 as adults; emerged en route from eggs of *Homalodisca liturata* on leaves of *C. canadensis*, S&R # 07–29) [11 specimens of both sexes, UCRC]. Also vouchered were 4 specimens [UCRC] of the first generation progeny of the aforementioned original wasps (emerged 6–8.viii.2007).

###### Comments

The taxonomy of the trichogrammatid genus *Pseudoligosita* Girault is in flux, and therefore further identification of this species was unwarranted. Morphologically, it is at least superficially indistinguishable from the undetermined species of *Pseudoligosita* from Tucumán, Argentina, an egg parasitoid of the proconiine sharpshooter *Tapajosa rubromarginata* (Signoret) (Jones 2001 [as *Oligosita* sp.]; Logarzo et al. 2004 [as one of the two *Oligosita* spp. mentioned]). The latter was identified as *Oligosita* sp. before Pinto and Viggiani (2004) renewed the status of *Pseudoligosita* as a genus separate from *Oligosita* Walker.

*Pseudoligosita* sp. is being reared under laboratory conditions in the UCR quarantine facility on *H. vitripennis* eggs on *E. japonica* leaves. This appears to be the first case in which a species of *Pseudoligosita* is reared in the laboratory on a proconiine sharpshooter host. At the time when this communication was written (late September 2007), the colony in the UCR quarantine facility successfully reached the F2 generation (the developmental period from exposure to the host eggs to emergence of the next generation wasps was 27 to 29 days at 22–23°C). *Pseudoligosita* sp. is a gregarious parasitoid, producing two to four adults per each egg of *H. vitripennis*.

##### *Ufens ceratus* Owen

Material examined: MEXICO, Sonora: “Campo La Esperanza” (nr. Miguel Alemán): 14.vi.2007, J. Bernal (emerged 26.vi-5.vii.2007 in UCR quarantine from eggs of *Homalodisca liturata* on leaves of *H. annuus*, S&R # 07–26) [numerous females and 2 males, UCRC]; 2.vii.2007, A. Fú-Castillo (arrived to UCR quarantine 10.vii.2007 as adults; emerged en route from eggs of *Homalodisca liturata* on leaves of *C. canadensis*, S&R # 07–29) [7 females, UCRC]. “Campo Nuevo” (nr. Miguel Alemán), 3.iv.2007, J. Bernal (emerged 12–13.iv.2007 in UCR quarantine from eggs of *Homalodisca liturata* on jojoba leaves, S&R # 07–19) [numerous females and males, UCRC].

###### Comments

*Ufens ceratus* is a common parasitoid of *H. liturata* eggs on jojoba in southern California, USA, and also of other proconiine sharpshooters in Mexico and the USA (Al-Wahaibi et al. 2005). It was treated as *Ufens* sp. by Triapitsyn et al. (2002) (specimens from Nuevo León and Tamaulipas, Mexico) and Triapitsyn (2003) (specimens from Florida, USA).

##### *Ufens* *principalis* Owen

Material examined: MEXICO, Sonora: “Campo La Esperanza” (nr. Miguel Alemán), 14.vi.2007, J. Bernal (emerged 28.vi.2007 in UCR quarantine from eggs of *Homalodisca liturata* on leaves of *H. annuus*, S&R # 07–26) [2 females, UCRC]. “Campo Nuevo” (nr. Miguel Alemán), 3.iv.2007, J. Bernal (emerged 12–13.iv.2007 in UCR quarantine from eggs of *Homalodisca liturata* on jojoba leaves, S&R # 07–19) [numerous females and males, UCRC].

###### Comments

*Ufens principalis* is another common egg parasitoid of *H. liturata* on jojoba in southern California, USA, and of *H. vitripennis* in California, and is also known from Arizona and New Mexico, USA (Al-Wahaibi et al. 2005). This species is here recorded for the first time from Mexico (Sonora).

### Editor's Note

Paper copies of this article will be deposited in the following libraries. Senckenberg Library, Frankfurt Germany; National Museum of Natural History, Paris, France; Field Museum of Natural History, Chicago, Illinois USA; the University of Wisconsin, Madison, USA; the University of Arizona, Tucson, Arizona USA; Smithsonian Institution Libraries, Washington D.C. U.S.A.; The Linnean Society, London, England.

## Abbreviations

UCR: University of California, Riverside
S&R: Shipping and Receiving (number)
UCRC: Entomology Research Museum, University of California, Riverside
F: antennaL funicular segment (of females) or antennal flagellar segment (of males)

## References

[bibr01] Al-Wahaibi AK, Owen AK, Morse JG (2005). Description and behavioural biology of two *Ufens* species (Hymenoptera: Trichogrammatidae), egg parasitoids of *Homalodisca* species (Hemiptera: Cicadellidae) in southern California.. *Bulletin of Entomological Research*.

[bibr02] de León JH, Morgan DJW (2007). Evaluation of molecular markers for discriminating *Gonatocerus morrilli* (Hymenoptera: Mymaridae): a biological control agent for *Homalodisca vitripennis*.. *Annals of the Entomological Society of America*.

[bibr03] George JN (2007). Review of the species of *Ittys* (Chalcidoidea: Trichogrammatidae) occuring in the United States, with the description of four new species.. *Zootaxa*.

[bibr04] Gibson GAP, Gibson GAP, Huber JT, Woolley JB (1997). Morphology and terminology..

[bibr05] Hoddle MS, Triapitsyn SV, Tariq MA, Oswalt S, Blincoe P, Ba A, Lorick T, Esser T (2004). Searching for and collecting egg parasitoids of the glassy-winged sharpshooter and other *Homalodisca* species in southeastern and southwestern Mexico.. *Proceedings of the 2004 Pierce's Disease Research Symposium*.

[bibr06] Hoddle MS, Triapitsyn SV, Tariq MA, Blincoe P, Mochel M, Oswalt S, Esser T (2005). Maintaining and evaluating quarantine cultures of *Gonatocerus* spp., promising egg parasitoids from Argentina and Mexico, for the classical biological control of the glassy-winged sharpshooter in California.. *Proceedings of the 2005 Pierce's Disease Research Symposium*.

[bibr07] Huber JT (1988). The species groups of *Gonatocerus* Nees in North America with a revision of the *sulphuripes* and *ater* groups (Hymenoptera: Mymaridae).. *Memoirs of the Entomological Society of Canada*.

[bibr08] Jones WA, Tariq MA, Oswalt S, Esser T (2001). Classical biological control of the glassy-winged sharp-shooter.. *Proceedings of the Pierce's Disease Research Symposium*.

[bibr09] Legner EF, Bellows TS, Bellows TS, Fisher TW (1999). Exploration for natural enemies.. *Handbook of biological control*.

[bibr10] Logarzo GA, Virla EG, Triapitsyn SV, Jones WA (2004). Biology of *Zagella delicata* (Hymenoptera: Trichogrammatidae), an egg parasitoid of the sharpshooter *Tapajosa rubromarginata* (Hemiptera: Clypeorrhyncha: Cicadellidae) in Argentina.. *Florida Entomologist*.

[bibr11] MacGregor R, Gutiérrez O (1983). *Guía de insectos nocivos para la agricultura en México*..

[bibr12] Pacheco Mendívil F (1985). Plagas de los cultivos agrícolas en Sonora y Baja California. *Libro Técnico No. 1*..

[bibr13] Pilkington LJ, Irvin NA, Boyd EA, Hoddle MS, Triapitsyn SV, Carey BG, Jones WA, Morgan DJW (2005). Biological control of glassywinged sharpshooter in California.. *California Agriculture*.

[bibr14] Pinto JD (2006). A review of the New World genera of Trichogrammatidae (Hymenoptera).. *Journal of Hymenoptera Research*.

[bibr15] Pinto JD, Viggiani G (2004). A review of the genera of Oligositini (Hymenoptera: Trichogrammatidae) with a preliminary hypothesis of phylogenetic relationships.. *Journal of Hymenoptera Research*.

[bibr16] Sorensen SJ, Gill RJ (1996). A range extension of *Homalodisca coagulata* (Say) (Hemiptera: Clypeorrhyncha: Cicadellidae) to southern California.. *Pan-Pacific Entomologist*.

[bibr17] Takiya D (2006). Sharpshooter (Cicadellinae) database search.. http://ctap.inhs.uiuc.edu/takiya/search.asp?key=Proconia&lng=En.

[bibr18] Triapitsyn SV (2003). Taxonomic notes on the genera and species of Trichogrammatidae (Hymenoptera)-egg parasitoids of the proconiine sharpshooters (Hemiptera: Clypeorrhyncha: Cicadellidae: Proconiini) in southeastern USA.. *Transactions of the American Entomological Society*.

[bibr19] Triapitsyn SV (2006a). A key to the Mymaridae (Hymenoptera) egg parasitoids of proconiine sharpshooters (Hemiptera: Cicadellidae) in the Nearctic region, with description of two new species of *Gonatocerus*.. *Zootaxa*.

[bibr20] Triapitsyn SV, Esser T, Tariq MA, Medeiros R, Mochel M, Veling S (2006b). Identify the species of Mymaridae reared in Argentina and Mexico for potential introduction to California against the glassy-winged sharpshooter and prepare and submit for publication a pictorial, annotated key to the *ater*-group species of *Gonatocerus*-egg parasitoids of the proconiine sharpshooters (Hemiptera: Cicadellidae: Proconiini) in the Neotropical region.. *Proceedings of the 2006 Pierce's Disease Research Symposium*.

[bibr21] Triapitsyn SV, Bezark LG, Morgan DJW (2002). Redescription of *Gonatocerus atriclavus* Girault (Hymenoptera: Mymaridae), with notes on other egg parasitoids of sharpshooters (Homoptera: Cicadellidae: Proconiini) in northeastern Mexico.. *Pan-Pacific Entomologist*.

[bibr22] Triapitsyn SV, Hoddle MS, Tariq MA, Oswalt S, Esser T (2001). Search for and collect egg parasitoids of glassy-winged sharpshooter in southeastern USA and northeastern Mexico.. *Proceedings of the 2001 Pierce's Disease Research Symposium*.

[bibr23] Triapitsyn SV, Hoddle MS, Tariq MA, Oswalt S, Blincoe P, Esser T (2002). Search for and collect egg parasitoids of glassy-winged sharpshooter in southeastern USA and northeastern Mexico.. *Proceedings of the 2002 Pierce's Disease Research Symposium*.

[bibr24] Triapitsyn SV, Phillips PA (2000). First host record of Gonatocerus triguttatus (Hymenoptera: Mymaridae) from eggs of Homalodisca coagulata (Homoptera: Cicadellidae), with notes on the distribution of the host.. *Florida Entomologist*.

[bibr25] Triapitsyn SV, Vickerman DB, Heraty JM, Logarzo GA (2006). A new species of Gonatocerus (Hymenoptera: Mymaridae) parasitic on proconiine sharpshooters (Hemiptera: Cicadellidae) in the New World.. Zootaxa.

[bibr26] Wiedenmann RN, Smith JW (1997). Novel associations and importation biological control: the need for ecological and physiological equivalencies.. *Insect Science and its Application*.

